# MicroRNA Transcriptome Profile Analysis in Porcine Muscle and the Effect of miR-143 on the MYH7 Gene and Protein

**DOI:** 10.1371/journal.pone.0124873

**Published:** 2015-04-27

**Authors:** Jianjun Zuo, Fan Wu, Yihua Liu, Juan Xiao, Mei Xu, Qinping Yu, Minhao Xia, Xiaojun He, Shigeng Zou, Huize Tan, Dingyuan Feng

**Affiliations:** 1 College of Animal Science of South China Agricultural University, Guangzhou, 510642, China; 2 Production Technology Department of WENs Group, Xinxing, 527400, China; National University of Singapore, SINGAPORE

## Abstract

Porcine skeletal muscle fibres are classified based on their different physiological and biochemical properties. Muscle fibre phenotype is regulated by several independent signalling pathways, including the mitogen-activated protein kinase (*MAPK*), nuclear factor of activated T cells (*NFAT*), myocyte enhancer factor 2 (*MEF2*) and peroxisome proliferator-activated receptor (PPAR) signalling pathways. MicroRNAs are non-coding small RNAs that regulate many biological processes. However, their function in muscle fibre type regulation remains unclear. The aim of our study was to identify miRNAs that regulate muscle fibre type during porcine growth to help understand the miRNA regulation mechanism of fibre differentiation. We performed Solexa/Illumina deep sequencing for the microRNAome during 3 muscle growth stages (63, 98 and 161 d). In this study, 271 mature miRNAs and 243 pre-miRNAs were identified. We detected 472 novel miRNAs in the muscle samples. Among the mature miRNAs, there are 23 highest expression miRNAs (over 10000 RPM), account for 85.3% of the total counts of mature miRNAs., including 10 (43.5%) muscle-related miRNAs (ssc-miR-133a-3p, ssc-miR-486, ssc-miR-1, ssc-miR-143-3p, ssc-miR-30a-5p, ssc-miR-181a, ssc-miR-148a-3p, ssc-miR-92a, ssc-miR-21, ssc-miR-126-5p). Particularly, both ssc-miR-1 and ssc-miR-133 belong to the MyomiRs, which control muscle myosin content, myofibre identity and muscle performance. The involvement of these miRNAs in muscle fibre phenotype provides new insight into the mechanism of muscle fibre regulation underlying muscle development. Furthermore, we performed cell transfection experiment. Overexpression/inhibition of ssc-miR-143-3p in porcine skeletal muscle satellite cell induced an/a increase/reduction of the slow muscle fibre gene and protein (*MYH7*), indicating that miR-143 activity regulated muscle fibre differentiate in skeletal muscle. And it regulate *MYH7* through the *HDAC4-MEF2* pathway.

## Introduction

Muscle fibre type is determined by the myosin structure or physiological capabilities of the muscle[1]. The composition of muscle fibres determines the muscle’s physicochemical properties, including fibre area, fibre density, oxidation capacity, and glycolysis capacity. Therefore, the muscle fibre composition is an important factor influencing many of the peri- and post-slaughter biochemical processes, thus influencing meat quality[2]. Our previous studies have indicated that meat colour, water holding capacity and meat pH are related to fibre composition. The regulation of fibre composition is a viable pathway for improving meat quality[3]. Based on the relative contraction speed of the fibre and its oxidative and glycolytic metabolism capacities, porcine muscle fibre has been classified into slow-twitch oxidative fibre (type 1), fast-twitch oxidative fibre (type 2a), fast-twitch glycolytic fibre (type 2b) and fast-twitch oxidative glycolytic fibre (type 2x)[4]. In postnatal porcine skeletal muscle, the number of muscle fibres is constant during growth. However, the type of muscle fibre can be regulated by various conditions, such as neuromuscular activity, mechanical loading, mechanical unloading, hormones, aging, and endurance exercise[5].

MicroRNAs are small non-coding RNAs molecular (approximately 22 nucleotides), which transcribed by RNA polymerase II. After transcription, the primary miRNA (pri-miRNA) transcripts, which are several hundred nucleotides in length, are cleaved to 60–80 nucleotides precursor miRNAs (pre-miRNAs). Pre-miRNAs are subsequently cleaved to form mature miRNAs, which join the RNA-induced silencing complex (RISC)[6]. Based on the base-pairing of their seed sequence complementary sequences within mRNAs, miRNAs play regulatory roles in many important physiological processes and have been demonstrated to be involved in mediating key aspects of skeletal muscle development and responses to diseases[7]. Mmu-miR-208 and mmu-miR-499 are encoded by the mouse Myh7 and Myh7b genes. These miRNAs are part of the myomiR network, which regulates the expression of the myosin heavy chain (*MYH*) genes[8]. The *MYH* genes family encodes a subunit of myosin, which is the motor protein of muscle thick filaments. Isoforms of myosin heavy chain (MyHC) have distinct characteristics necessary for defining specific types of muscle fibre[9]. Innervation-regulated signalling cascades in skeletal muscle control the activation of downstream transcription factors to regulate the expression of fibre type-specific myosin genes. A previous study on avian skeletal muscle fibres has shown that the expression of *MEF2* and *NFAT* was required for innervation-induced expression of slow myosin heavy chain 2 (*MYH2*). The gene upstream of *MYH2* contains binding sites for *MEF2* and *NFAT*[10]. *MEF2* is an important factor for muscle development and fibre differentiation. Previous reports have indicated that *MEF2* could activate the expression of miR-92b, which then downregulates *MEF2* through binding to its 3’UTR. A negative feedback circuit exists between miR-92b and *MEF2*[11]. Endurance exercise training can increase the proportion of type 1 fibre, which mainly use oxidative metabolism for energy production and are more fatigue-resistant. The mechanism of this transformation is the training-induced targeted expression of an activated form of PPARδ, which induces increase in oxidative enzymes, mitochondrial biogenesis and the number of type 1 muscle fibres[12]. PPARδ is the common target of the microRNA cluster miR-199a~214. An antagomir-based silencing experiment suggested that the miR-199a~214 cluster actively represses cardiac PPARδ expression[13]. There are links between miRNAs and the signalling pathways that regulate muscle fibre composition. However, research into the regulation of muscle fibre type by miRNAs is still rare.

The growth process is associated with an increasing muscle mass and the transition of muscle fibre type[14]. A previous study in cattle showed that skeletal muscle fibre type 2a transforms into type 2b in the first months after birth and that skeletal muscle fibre type 1 is nearly unaffected by age[15]. There are many factors that influence the composition of skeletal muscle fibres, and there are also many ways to regulate the fibre type, including endurance training, nutrition, gene interference, and miRNAs. Previous studies on muscle fibre regulation have focused on the period before weaning. However, the composition of muscle fibre changes after weaning and throughout growth.

The aim of this study was to investigate the transformation of muscle fibre type during the period from 35 to 161 d and specifically to identify the miRNAs that play key roles in the regulation of muscle fibre type during this period.

## Results and Discussion

### Content of different muscle fibres at 63, 98 and 161 d

The psoas major muscle at 63, 98 and 161 d was isolated to analyse the composition of muscle fibres. Fibres were classified into 3 types (1, 2a, and 2x+2b) using enzyme histochemical staining based on the acid stability of myosin ATPase and the glycolytic rate (Table 1). During the porcine growing period, the proportion of type 1 fibres decreased significantly from 9.21 to 4.21%, and the proportion of white fibres (2x+2b) increased significantly from 63 d to 98 and 161 d.

**Table 1:** Composition of the psoas major muscle fibres at 63, 98 and 161 d.

Muscle fibre type (%)	63 d	98 d	161 d
**Fibre 1**	***9*.*21±0*.*79*** ^***a***^	***6*.*03±0*.*76*** ^***b***^	***4*.*21±0*.*44*** ^***c***^
**Fibre 2a**	19.08±1.75	16.20±1.25	16.44±1.49
**Fibre 2x+2b**	***71*.*71±1*.*79*** ^***a***^	***77*.*78±1*.*56*** ^***b***^	***79*.*35±1*.*72*** ^***b***^

^a, b, c^ Data with different superscripts in the same row are significantly different (P<0.05).

All fibres were initially red fibre types (type 1 and type 2a) in new born piglets. Fibre type 2a has the capacity to transform from a red fibre to a white fibre (mainly to fibre type 2x). Transformation of inactive muscle, such as the pectoralis of chick, occurs much more easily than transformation of active muscle[16]. In our study, red fibres (type 1 and type 2a) also transformed to white fibres (type 2x and type 2b) in the period from 63 to 161 d. However, the variation rate and speed of the muscle fibre type was less than in the period before weaning. Fibre type transformation followed a regular sequence as follows: 1↔2a↔2x↔2b[5]. The decline in the proportion of type 1 fibres in the period from 63 to 161 d indicated that some fibres of type 1 transformed to type 2a and that some fibres of type 2a transformed to type 2x during this period. Furthermore the muscle’s capacity for oxidative metabolism will decline with the reduction in red fibres. The meat colour, one component of the meat quality index, will also change following the changes in muscle fibre composition[17]. To determine whether the apparent variation in these traits is caused by the differential expression of genes in the *MYH* genes family, which encoded proteins characteristic of different types of muscle fibre, we used RT-qPCR to assess the expression of *MYH* family at 63d, 98d and 161d.

### Expression of *MYH* genes at 63, 98 and 161 d

Myosin II is the type of myosin responsible for producing contractions in muscle cell. There are 16 heavy chains and 13 light chains for myosin, each of which has different functional and physicochemical properties[18]. The myosins of different muscle fibres are composed of different myosin heavy chains and light chains. The porcine myosin heavy chains have four characteristic isoforms (1, 2a, 2x, and 2b) in skeletal muscle, and they are encoded by a family of genes (*MYH7*, *MYH2*, *MYH1*, and *MYH4*), that are used to identify different muscle fibres. In most fibres, the composition of transcripts is closely related to the composition of the corresponding fibres[4].

**Fig 1:**
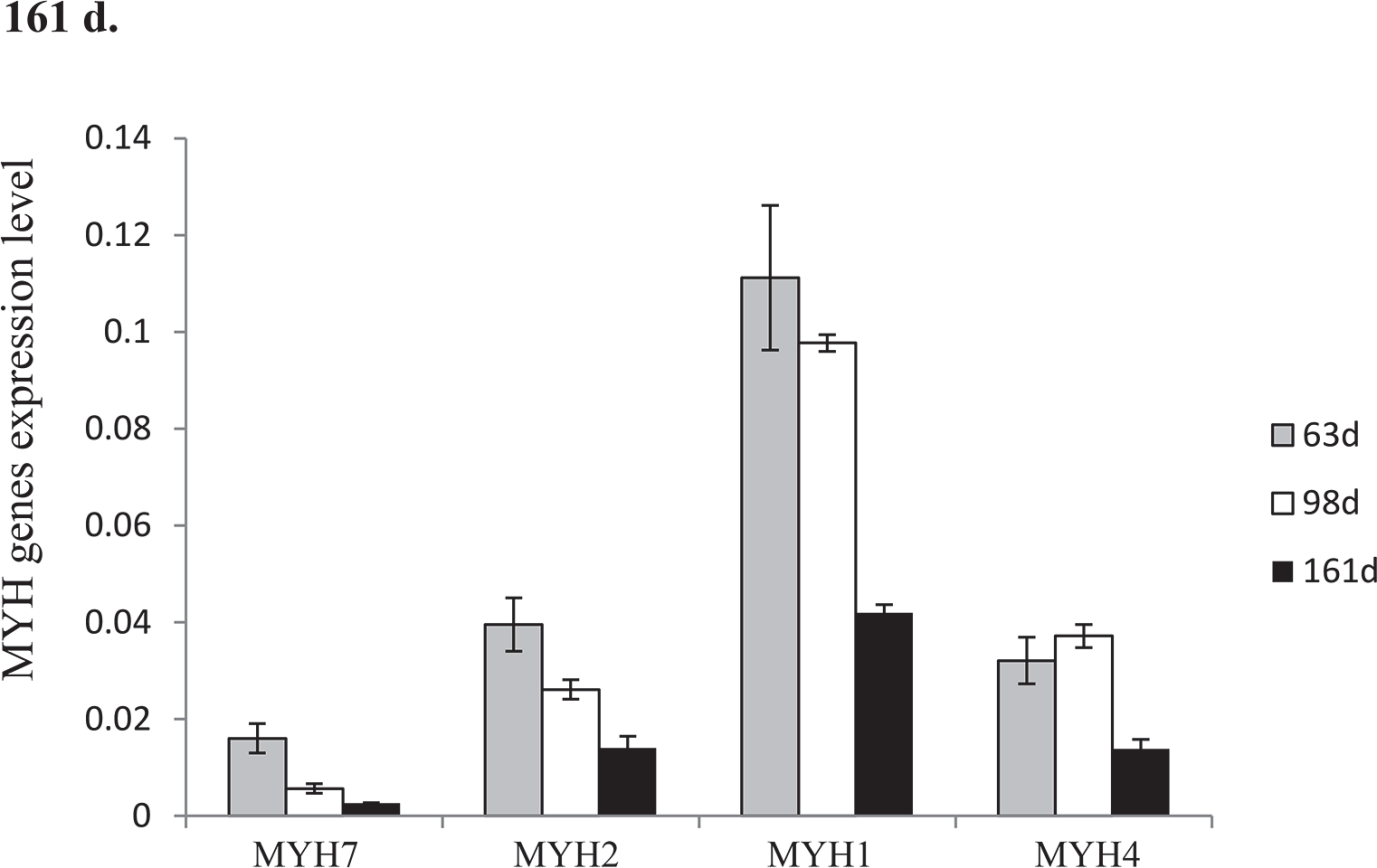
MyHC gene expression in the psoas major muscle at 63, 98 and 161 d.

The expression levels of *MYH* genes in the psoas major muscle were determined by real-time qPCR (Fig 1). *MYH7* (type 1) and *MYH2* (type 2a) were both significantly decreased in the period from 63 to 161 d. *MYH1* (type 2x) and *MYH4* (type 2b) were significantly decreased in the last development stage (p<0.05).

The composition of muscle fibres determined by real-time qPCR showed the trends similar to those of the composition determined by staining. This indicates that there are high positive correlations between the two methods of muscle fibre typing. Furthermore, the expression levels of *MYH* family genes are directly related to the apparent traits of different muscle fibres. However, the expression level of MyHCs showed an overall decreasing trend during the period from 63d to 161d indicating that there may be endogenous factors playing key roles to induce the downregulation of *MYH* genes during this period. For example, myoblasts from presumptive fast and slow muscles may be programmed to express and suppress different MyHC subtypes[19].

### Characterisation of miRNA expression profiles in the porcine psoas major muscle at 63, 98 and 161 d as determined by deep sequencing

**Fig 2:**
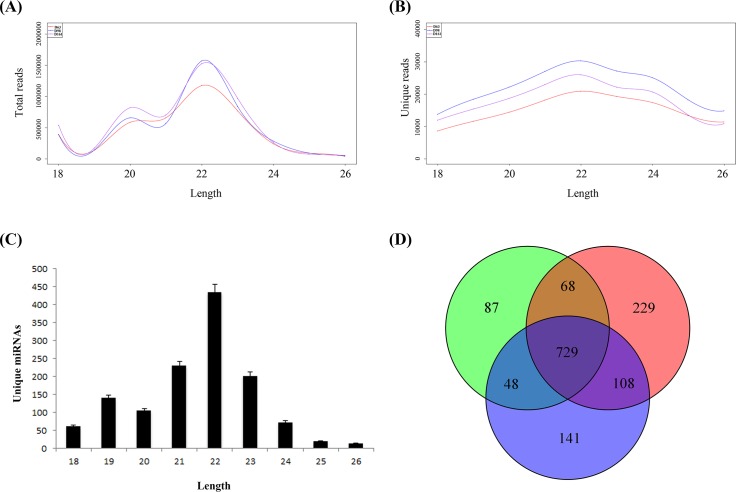
Basic information from the sequencing data. (A) Length distribution of the total reads; (B) Length distribution of the unique reads; (C) Length distribution of the unique miRNAs; (D) The numbers of detected miRNAs in the three libraries. (p<0.05).

To discover the reason behind the changes in muscle fibre composition, Solexa/Illumina deep sequencing was used to examine the miRNA expression profiles of porcine psoas major muscle at 63, 98 and 161 d. After eliminating the common RNA, such as mRNA, RFam (rRNA, tRNA, snoRNA and snRNA), repeats and other unknown small RNA, the remaining total clean read count for the muscle sample at 63, 98 and 161 d exceeded 4,100,000, 4,600,000 and 5,100,000, respectively. The reads from the raw data to cleaned sequences are shown in S1 Table. There were 135,469, 197,281 and 162,541 cleaned unique reads among the total reads at 63, 98, and 161 d, accounting for 26.47%, 28.91% and 29.06% of the unique reads, respectively. The most abundant size of small RNA detected was 22 nt, followed by 20, 21 and 23 nt (Fig 2A, 2B and 2C).

Comparing the data to miRBase Release 20.0, there were 887 sequences associated with known miRNAs. Among them, 271 (96.8%) porcine mature miRNAs and 243 (88.0%) porcine pre-miRNAs were identified, and we detected 472 novel miRNAs. There were 729 common expression sequences at the three stages tested, and 87, 229 and 141 miRNAs with specific expression at 63d, 98d and 161d, respectively (Fig 2D). However, all the miRNAs with specific expression at the various stages were expressed at very low levels. All of the above results indicated that the major clean reads mapped to known miRNAs in miRBase and highly were enriched for porcine sequence data, suggesting that the deep sequence data are representative of the miRNA expression profile of porcine skeletal muscle and would be reliable for the subsequent analyses and prediction of novel miRNAs.

Solexa deep sequencing is an advanced technique for the discovery of novel miRNAs. In our study, we defined novel miRNAs as being 18 to 26 nt in length and not identifiable from previously reported sequences. After filtering, we obtained 472 novel miRNAs and named them PC-N (N = 1~472) in S4 Table. The novel miRNAs were generally expressed at very low levels. Among the novel miRNAs, total normalised expression counts from the three libraries below 100 RPM was observed for 463, accounting for 98.1% of the total novel miRNAs. Furthermore, only one miRNA showed total expression over 1000 RPM. This result is similar to that of a previous study[20]. Because of their low expression levels, we eliminated most of the novel miRNAs from the subsequent analyses.

### Most abundant miRNAs at different stages

A previous study indicated that miRNAs detected by deep sequencing that were expressed below 100–1,000 reads per million (RPM) had no discernible activity[21]. We summarised the distribution of the normalised expression of 271 porcine mature miRNAs in S3 Table. The numbers of miRNAs with normalised expression below 1000 RPM are 203, 200 and 200 at the three stages tested, accounting for 74.9%, 73.8% and 73.8% of the total mature miRNAs, respectively. There were 48, 50 and 51 miRNAs with mean expression between 1,000 and 10,000 RPM, accounting for 17.7%, 18.5% and 18.8% of the total mature miRNAs, respectively. However, the numbers of miRNAs with mean expression over 10000 RPM at three stages are 20, 21 and 20, account for 7.4%, 7.7% and 7.4%, respectively. The most abundant miRNAs in each library are listed in Table 2. Furthermore, the 23 miRNAs with the highest expression overall account for 85.3% of the total counts of all counts of these 271 porcine miRNAs. This result indicates that the majority of clean reads mapped to few miRNAs, which is also consistent with a previous study[22].

**Table 2:** Most abundant miRNAs in skeletal muscle (RPM>10000).

miR name	D63	D98	D161	Total counts	Percent
**ssc-miR-133a-3p**	408806	315123	477166	1201095	32.6%
**ssc-miR-26a**	69941	105652	103735	279328	7.6%
**ssc-miR-486**	77118	70149	92245	239512	6.5%
**ssc-miR-10b**	52416	81007	92091	225515	6.1%
**ssc-miR-1**	24349	57426	47962	129736	3.5%
**ssc-let-7a**	41744	36695	32087	110526	3.0%
**ssc-miR-378**	47265	24873	27620	99758	2.7%
**ssc-miR-143-3p**	17551	20769	59773	98093	2.7%
**ssc-miR-30e-5p**	24314	36213	36339	96866	2.6%
**ssc-miR-30a-5p**	25090	28670	32484	86244	2.3%
**ssc-miR-27b-3p**	22193	23111	26313	71617	1.9%
**ssc-miR-30d**	21509	19996	21639	63144	1.7%
**ssc-let-7f**	17073	23841	17433	58347	1.6%
**ssc-miR-191**	15771	18887	19525	54183	1.5%
**ssc-miR-181a**	18027	11681	19158	48866	1.3%
**ssc-miR-148a-3p**	8851	14004	18387	41242	1.1%
**ssc-miR-92a**	20096	9231	9855	39181	1.1%
**ssc-let-7g**	13180	14608	10799	38587	1.0%
**ssc-let-7i**	15727	11228	9664	36618	1.0%
**ssc-let-7c**	10980	10202	10232	31414	0.9%
**ssc-miR-21**	9124	11801	10477	31402	0.9%
**ssc-miR-126-5p**	7187	12467	10788	30443	0.8%
**ssc-miR-127**	13523	6544	6353	26419	0.7%

The muscle-specific myosin heavy chain genes encode a family of miRNAs, called myomiRs, which control muscle performance, myofibre identity and myosin content[23]. Among the 23 miRNAs with the highest expression, there are 10 (43.5%) muscle-related miRNAs (ssc-miR-133a-3p, ssc-miR-486, ssc-miR-1, ssc-miR-143-3p, ssc-miR-30a-5p, ssc-miR-181a, ssc-miR-148a-3p, ssc-miR-92a, ssc-miR-21, ssc-miR-126-5p). Notably, the most abundant ssc-miR-133a-3p and ssc-miR-1, are myomiRs. They clustered on the same chromosomal loci and have been shown to modulate skeletal muscle proliferation and differentiation at the cell and embryo levels[24]. However, their mechanisms of regulation are not the same. MiR-1 regulates muscle growth by targeting histone deacetylase 4 (*HDAC4*), which represses an essential muscle-related transcription factor (myocyte enhancer factor 2C, *MEF2C*)[24]. *MEF2* proteins in cooperation with *PGC-1α* play an important role in regulating oxidative metabolism and synergistically activating the slow-twitch fibre gene[25]. Furthermore, miR-133 enhances the proliferation of myoblasts by repressing the expression of serum response factor (*SRF*)[24]. MiR-486, the second-most abundant miRNA has been identified as a downstream mediator of myocardin-related transcription factor-A (*MRTF-A*), serum response factor (*SRF*) and *MyoD*[26]. The remaining myomiRs also participate in certain processes of muscle proliferation and differentiation as outlined in Table 3.

**Table 3:** The function of highly expressed muscle-related miRNAs in muscle development.

miRs	Targets	miRs-targets relationship
**miR-133a-3p**	SRF	down-regulation
**miR-486**	PTEN, FOXO1A	down-regulation
**miR-1**	HDAC4, PAX3, PAX7	down-regulation
**miR-143-3p**	SRF, myocardin, Nkx2-5	down-regulation
**miR-30a-5p**	PTC1, CTGF, SMO	down-regulation
**miR-181a**	Hox-A11	down-regulation
**miR-148a-3p**	ROCK1	down-regulation
**miR-92a**	MEF2	down-regulation
**miR-21**	WNT1, JAG1	down-regulation
**miR-126-5p**	VCAM-1	down-regulation

### Validation of miRNA expression by stem-loop RT-qPCR

**Fig 3:**
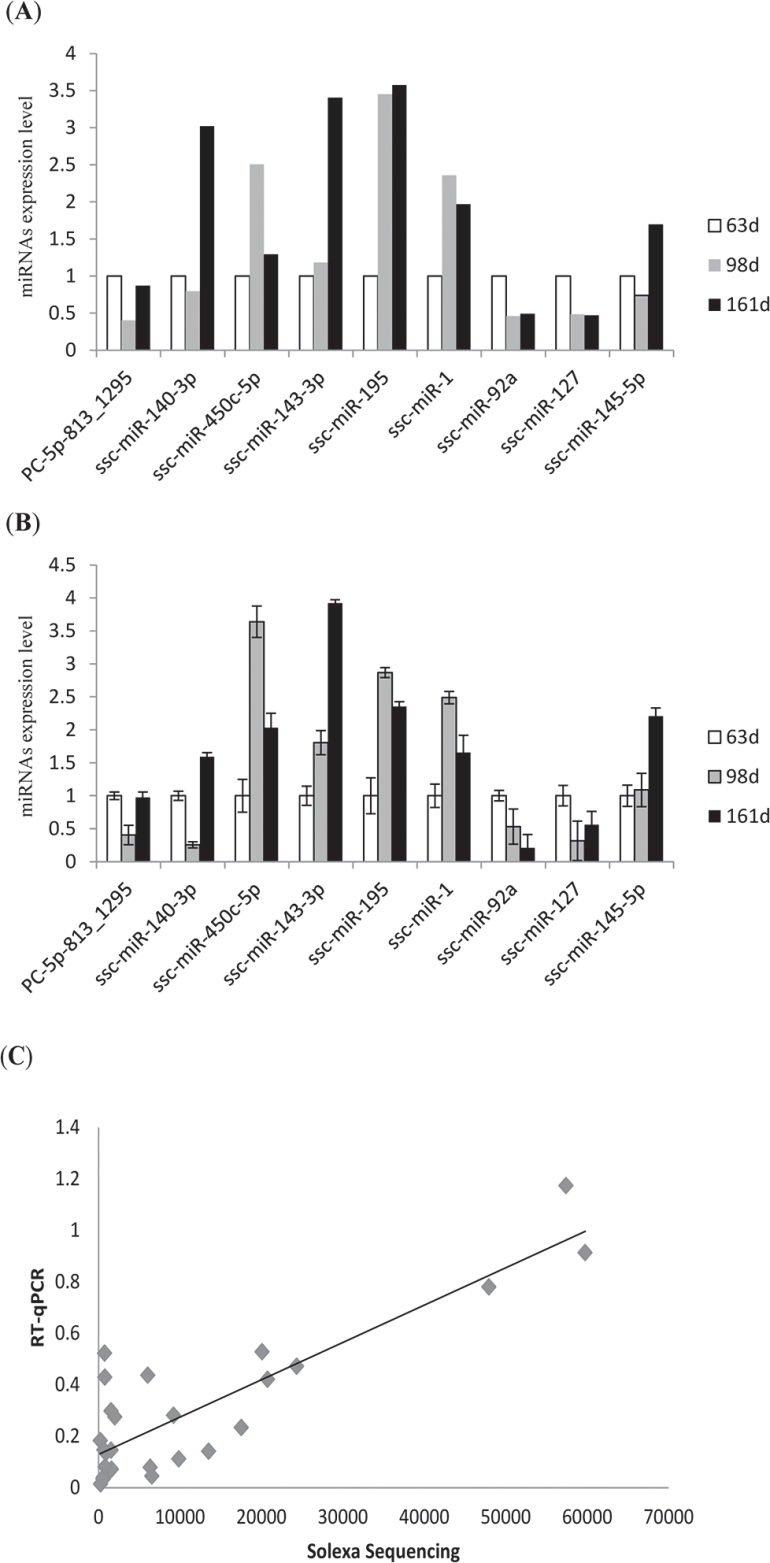
RT-PCR verification of miRNAs detected by solexa sequencing. (**A**) Fold change of differentially expressed miRNAs at 63, 98 and 161 d as determined by sequencing. (**B**) Fold change of differentially expressed miRNAs at 63, 98 and 161 d as determined by RT-qPCR. (**C**) Correlation coefficients (R) between miRNA expression profiles as determined by Illumina sequencing and RT-qPCR. The miRNAs selected for analysis are PC-5p-813_1295, ssc-miR-140-3p, ssc-miR-450c-5p, ssc-miR-143-3p, ssc-miR-195, ssc-miR-1, ssc-miR-92a, ssc-miR-127 and ssc-miR-145-5p.

To validate the miRNA expression profile determined by sequencing, we randomly selected 9 differentially expressed miRNAs for stem-loop RT-qPCR assay (Fig 3). All 9 miRNAs were detected by RT-qPCR at 63, 98 and 161 d (Fig 3B), and they closely matched the overall trends at the three stages as determined by sequencing (Fig 3A). The correlation coefficient between the two ethods was 0.86, indicating that the results obtained using the two methods were closely related (Fig 3C). These results further demonstrated that deep sequencing is a reliable technique for ascertaining the gene expression profile of animal muscle samples, and the sequencing results were considered reliable for subsequent analyses.

### Differential expression of miRNAs in different stages

The differentially expressed (DE) miRNAs may play important roles in biological processes. To identify typical miRNAs involved in the regulation of muscle fibre type, we compared the libraries of the three stages. After normalisation, a 2-fold change between groups was used to filter differentially expressed miRNAs (p<0.05). Using filter criteria, there were 89 (47 up/42 down), 64 (21 up/43 down) and 120 (48 up/72 down) DE miRNAs between 63d and 98d, 98d and 161d and 63d and 161d, respectively (Table 4). However, a previous study demonstrated that miRNAs identified by sequencing and expressed at low levels usually had no discernible activity[21]. The miRNAs with the normalised expression levels over 300 RPM are listed in Table 5. There were 4 upregulated miRNAs and 5 downregulated miRNAs between 63 and 98 d, 4 upregulated miRNAs and 1 downregulated miRNA between 98 and 161 d, and 5 upregulated miRNAs and 3 downregulated miRNAs between 63 and 161 d. Among these miRNAs, ssc-miR-1, ssc-miR-92a, ssc-miR-143-3p and ssc-miR-127 were highly expressed (normalised reads over 10000 RPM), and all 4 of these miRNAs are muscle-related. In particular, miR-1 is a myomiR. These miRNAs may therefore participate in muscle development and differentiation through various pathways.

**Table 4:** Summary of differential expression miRNAs between the three stages.

The comparison between libraries	DE miRNAs	upregulated miRNAs	downregulated miRNAs
D63 vs D98	89	47	42
D98 vs D161	64	21	43
D63 vs D161	120	48	72

DE: differential expression, fold change>2 or fold change<0.5, p<0.05.

**Table 5:** Differential expression of miRNAs between the three stages(expression level > 300 RPM).

miRs name	EL (D63)	EL (D98)	EL (D161)	FC(63vs98)	FC(98vs161)	FC(63vs161)	p-value
ssc-miR-1	24349	57426	47962	2.36	0.84	1.97	0
ssc-miR-127	13523	6544	6353	0.48	0.97	0.47	0
ssc-mir-1285-p5	1470	722	912	0.49	1.26	0.62	2.20E-56
ssc-miR-139-5p	665	377	327	0.57	0.87	0.49	3.66E-42
ssc-miR-140-3p	2007	1595	6058	0.79	3.80	3.02	0
ssc-miR-143-3p	17551	20769	59773	1.18	2.88	3.41	0
ssc-miR-145-5p	914	675	1552	0.74	2.30	1.70	6.84E-47
ssc-miR-148a-3p	8851	14004	18387	1.58	1.31	2.08	0
ssc-miR-181c	356	457	950	1.28	2.08	2.67	2.37E-22
ssc-miR-28-3p	2101	1013	1360	0.48	1.34	0.65	2.22E-83
ssc-miR-335	395	753	240	1.91	0.32	0.61	8.40E-86
ssc-miR-340	630	1236	2173	1.96	1.76	3.45	3.00E-142
ssc-miR-374a-5p	402	867	662	2.16	0.76	1.65	5.06E-41
ssc-miR-423-3p	5014	2407	3305	0.48	1.37	0.66	1.28E-198
ssc-miR-450b-5p	675	1781	1228	2.64	0.69	1.82	2.13E-117
ssc-miR-450c-5p	619	1550	801	2.50	0.52	1.29	1.92E-94
ssc-miR-92a	20096	9231	9855	0.46	1.07	0.49	0

EL: expression level, fold change > 2 or fold change < 0.5, expression level > 300 RPM, p<0.05.

For example, miR-1 regulates the expression of slow-twitch muscle fibre through the *HDAC4/MEF2* pathway. In our study, ssc-miR-1 was upregulated more than 2-fold in the period from 63 to 98 d suggesting that it may play a key role in the regulation of slow-twitch fibres during this period. A previous study demonstrated that mmu-miR-92a controls the growth of new blood vessels and may serve as valuable therapeutic target in the treatment of ischemic disease[27]. Other studies have also detected high expression levels of bta-miR-92a in skeletal muscle[28, 29]. This result indicates that miR-92a may be involved in the regulation of skeletal muscle growth. As predicted by TargetScan, miR-127 targets lysine methyltransferase 8 (*SETD8*), mitogen-activated protein kinase 4 (*MAPK4*) and aconitase 2 (*ACO2*). SETD8 plays a crucial role in lysine methylation[30], and lysine regulates muscle fibre type through its effect on *MEF2*[31]. The MAPK signalling pathway is involved in motor neurons and signalling systems. Activation of the MAPK pathway induces effects similar to those of slow motor neurons on the expression of myosin genes[32]. Thus, we speculated that miR-127 may regulate muscle fibre types via these three pathways. A previous mmu-miR-143 knockout experiment indicated that the expression of the mmu-miR-143 cluster is essential for vascular smooth muscle cells (VSMCs) to acquire the contractile phenotype[33]. MiR-143 is involved in adipocyte differentiation through target genes, including *ERK5*[34].Another study demonstrated that the expression of miR-143 was negatively correlated with the expression of *MyoD* in both fast and slow muscles of Siniperca chuatsi. Furthermore, the suppression of miR-143 induced notable upregulation of *MyoD* and fast *MYH* genes, indicating that miR-143 is involved in controlling the performance of different fibre types in vertebrate[35]. However, studies on miR-143 in porcine skeletal muscle are rare. In our study, miR-143-3p was upregulated from 63d to 161d, with a fold change between 98 d and 161 d greater than 2. Understanding whether the transformation of muscle fibre type is caused by the changes of ssc-miR-143-3p expression merits further research.

### miRNA target prediction and Kyoto Encyclopedia of Genes and Genomes (KEGG) pathways analysis

**Fig 4:**
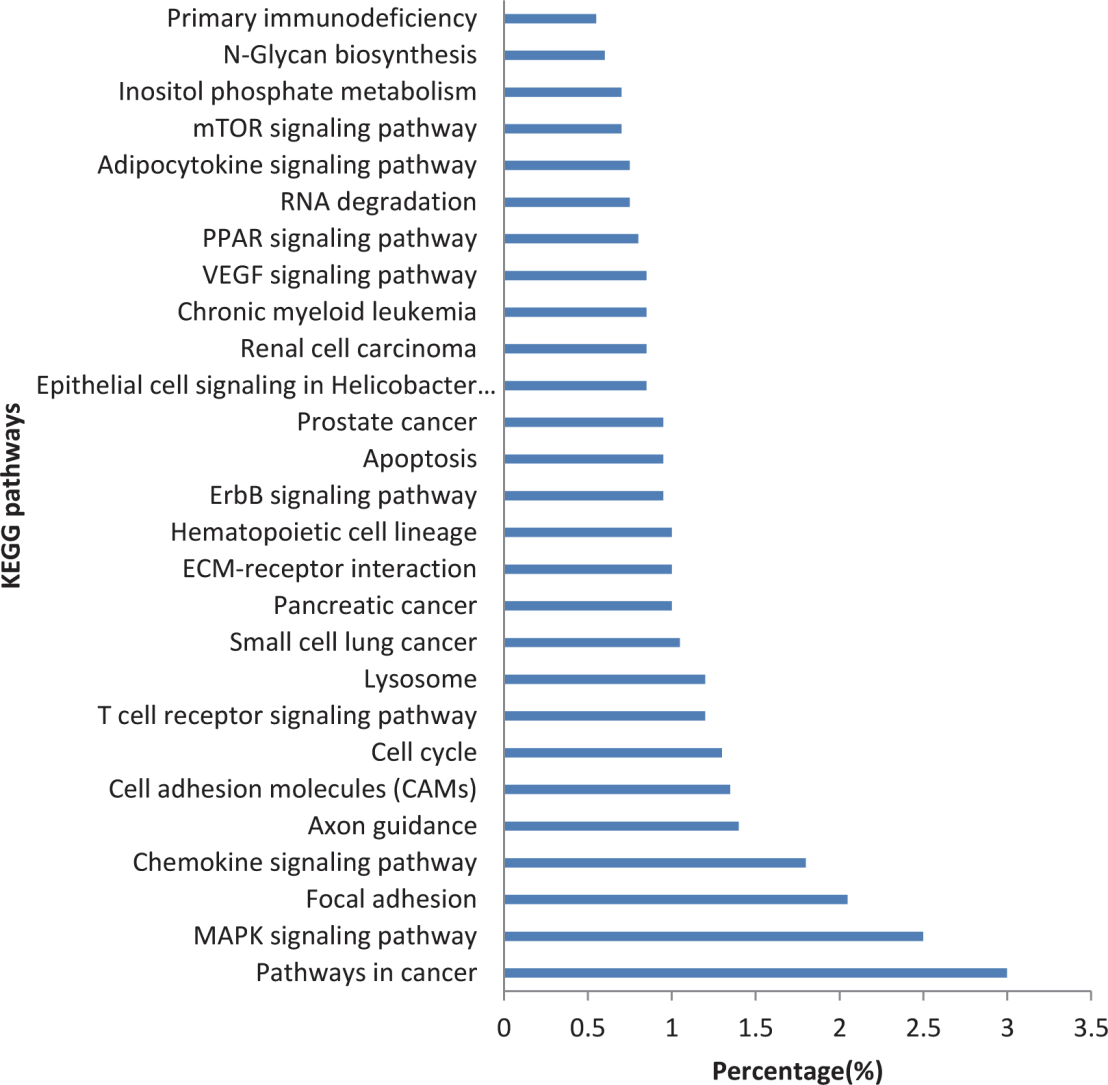
KEGG pathway analysis of most abundant miRNAs.

To further research the biological function of identified miRNAs, TargetScan and miRanda were used to predict the potential mRNA targets for the most abundant miRNAs (listed in Table 2) and highly differentially expressed (HDE) miRNAs (fold change >1.8 or <0.56; normalisation reads over 1000 RPM, listed in S5 Table). Because porcine genes are not included in the current TargetScan and miRanda databases, our predictions were based on human mRNAs. To analyse the functions of miRNAs involved in the muscle growth process, we selected targets of the abovementioned miRNAs and classified them according to the KEGG pathway enrichment analysis using DAVID functional annotation. A total of 2884 unique potential targets (2577 with name annotation) for the most abundant miRNAs (except the ubiquitously expressed let-7 family) were predicted. Twenty-seven enriched pathways were identified (p<0.05, Fig 4). These pathways are involved in disease, immunity, cell differentiate and communication. In particular, some enriched pathways were involved in the regulations of muscle fibre, including the MAPK[23], Peroxisome proliferator-activated receptors (PPAR)[12, 36], ErbB[37, 38] and mTOR[39] signalling pathways.

To identify the function of HDE miRNAs on the different biological process, we performed KEGG pathways analysis for the targets of HDE miRNAs at 63d to 98d, 98d to 161d and 63d to 161d. There were 17, 18 and 22 enriched pathways at different stages, respectively (p<0.05). And we summarized the pathways with the enriched genes over 1% of the total performed genes in S6 Table. There were 29 enriched pathways at the tree stages. Among them, PPAR, ErbB, Insulin, mTOR signalling pathways, adherens junction, and focal adhesion, were reported involved in the regulation of muscle proliferation and differentiation. Particularly, the PPAR signalling pathway were both highly enriched in the periods from 63 to 98 d and from 98 to 161 d. PPARs are nuclear hormone receptors that are activated by fatty acids and their derivatives. One of the PPAR subtypes, PPARδ, is involved in the regulation of muscle fibre type. Treatment of muscle with a PPARδ agonist induces an increasing number of type 1 fibres[12]. The PPARγ coactivator-1 (PGC-1) and PPARα may play a key role in inducing the increase in skeletal muscle mitochondrial content and the oxidative muscle fibre phenotype[36]. Thus, the PPAR signalling pathway may play an important role in muscle fibre regulation during this period.

### Integrated analysis of the mRNA and miRNA expression profiles

MiRNAs play an important role in RNA silencing and the post-transcriptional regulation of mRNA expression. In general, the expression patterns of miRNAs and their target mRNAs show opposing trends. We also performed mRNA Illumina deep sequencing for the psoas major muscle at different stages and an integrated analysis of the mRNA and the miRNA expression profiles. The miRNAs (normalisation reads over 100; a fold change greater than 1.8 or less than 0.56; p<0.05) and their target mRNAs (normalisation reads over 100; p<0.05) showing opposite expression trends are listed in S7 Table. The miRNAs marked in red colour were expressed at a high level (normalisation expression level over 10000 RPM), and the targeted genes shown in red, including *ACO2* [40], *PDK4*[41, 42], *CFL2*, *TNNC2*, *FLNC*[43], *ITM2B*, and *MYL1*, are reported involved as being in the regulation of muscle function. These miRNAs and mRNAs are crucial factors in muscle regulation. For instance, aconitate 2, encoded by the *ACO2* gene, is essential for lysine biosynthesis during filamentous fungal respiration [40]. In addition, studies have indicated that the *MEF2* gene may be regulated by lysine[44], and the *MEF2* binding site appears to be necessary for generating slow or oxidative muscle fibre[45]. Therefore, *ACO2* may regulate muscle fibre composition through its effects on lysine and MEF2. Pyruvate dehydrogenase kinase 4 (*PDK4*) is a master gene involved in muscle oxidative metabolism, and a study of the effect of two different exercises on different fibre types indicated that *PDK4* is significantly increased in both fibre types (type 1 and type 2) after prolonged exercise, with no difference between continuous exercise and interval exercise[41]. Many studies have demonstrated that exercise can increase the content of type 1 fibre, providing evidence that there are links between miRNAs and muscle fibre regulation. In particularly, FilaminC (*FLNC*), which is the potential target of the most abundant miR-143-3p, is one member of a family of actin binding proteins. A previous study on *FLNC* loss in vitro demonstrated that *FLNC* has a crucial role in muscle development and in the maintenance of muscle structural integrity[43]. And miR-143 also targets *HDAC4* and *MyoD*, which are reportedly involved in the regulation of muscle fibre types[35]. Therefore, miR-143-3p may regulate muscle fibre in a similar fashion.

### Effect of ssc-miR-143-3p on the slow fibre *MYH7* gene and protein expression in skeletal muscle satellite cells

**Fig 5:**
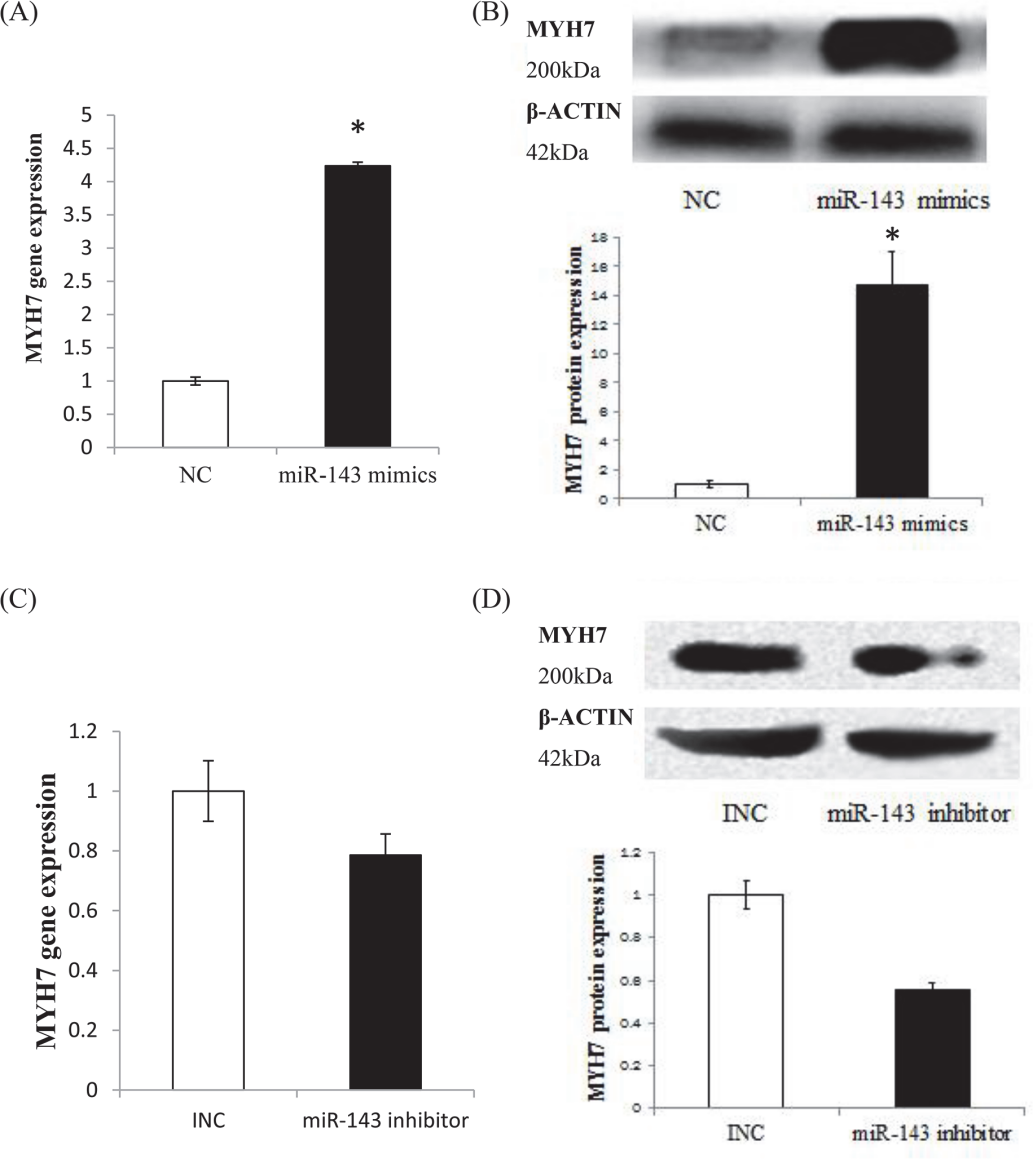
The expression levels of *MYH7* gene and protein in porcine skeletal muscle satellite cells after transferred ssc-miR-143 mimics and inhibitor. (A) The expression level of slow fibre gene *MYH7* after transferred ssc-miR-143-3p mimics; (B) The expression level of slow-twitch muscle myosin heavy chain protein after transfection with ssc-miR-143-3p mimics; (C) The expression level of slow fibre gene *MYH7* after transferred ssc-miR-143-3p inhibitor; (D) The expression level of slow-twitch muscle myosin heavy chain protein after transferred ssc-miR-143-3p inhibitor. NC is the control group for the miR-143 mimics transferred experiment. And INC is the control group for the miR-143 inhibitor transferred experiment. (*p<0.05, n = 5).

To gain further insight into the function of miR-143-3p on muscle differentiation, we transferred mimics of ssc-miR-143-3p into porcine skeletal muscle satellite cells (SCs), for ssc-miR-143-3p overexpression. The expression of the *MYH7* gene in SCs was significantly increased after transfection with ssc-miR-143-3p mimics (Fig 5A, p<0.05). To identify the effect of ssc-miR-143-3p on myosin protein, we performed western blotting (WB) using total protein extracted from the transfected cells. The results indicated that the protein encoded by the *MYH7* gene was also significantly increase following *MYH7* gene upregulation (Fig 5B, p<0.05). To obtain more evidence that slow fibres are regulated by ssc-miR-143-3p, we also transfected an ssc-miR-143-3p inhibitor into SCs, and the result was consistent with that for ssc-miR-143-3p mimic transfection. *MYH7* gene expression was repressed after ssc-miR-143-3p inhibitor transfection, though it was not significant (Fig 5C). In addition, the *MYH7* protein was significantly repressed by the ssc-miR-143-3p inhibitor (Fig 5D). Together, all of these results provide evidence that the ssc-miR-143-3p plays an important role in the regulation of slow muscle fibre in vitro.

**Fig 6:**
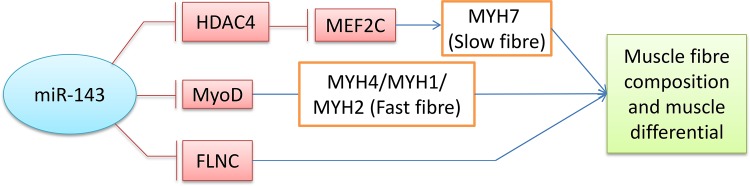
Potential regulation pathways of muscle fibres by ssc-miR-143-3p.

The upregulation/downregulation of ssc-miR-143-3p in skeletal muscle could induce the increase/repression of slow muscle fibres. However, the mechanism by which ssc-miR-143-3p regulates muscle fibres remains enigmatic. On the basis of the potential targets of ssc-miR-143-3p and previous studies, we inferred three possible pathways involved in the regulation of muscle fibres by ssc-miR-143-3p. First, miR-143 directly targets *MyoD*, crucial regulator of the fast fibre phenotype[46], repressing its expression by binding to the of *MyoD* 3’UTR[35, 46], and regulated fast fibre through *MyoD*. Second, miR-143 promotes slow fibres by targeting *HDAC4*, repressing its expression, and *HDAC4* inhibits slow muscle gene expression by repressing *MEF2C*, an essential transcription factor of muscle development[24]. Third, miR-143 also regulates muscle differentiation by targeting *FLNC*[43] (Fig 6). However, which pathway plays the major role in the muscle fibre regulation remains unclear. Our subsequent experiments aim to illustrate this regulation mechanism.

### Identify the pathways involved in the regulation of muscle fibres by ssc-miR-143-3p

**Fig 7:**
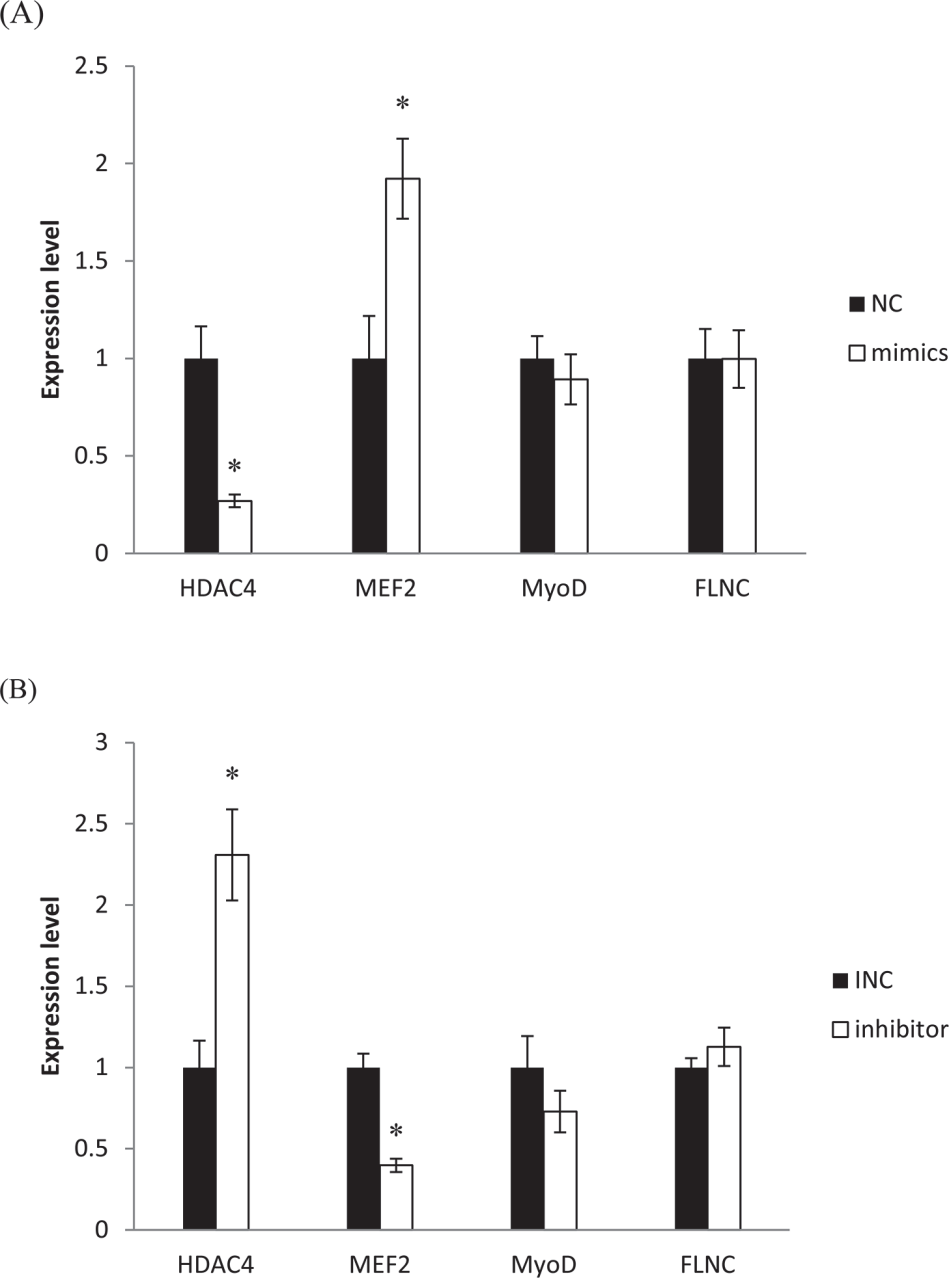
The expression levels of key genes in the potential regulation pathways after transferred ssc-miR-143 mimics and inhibitor. (A) The expression levels of key genes in the potential regulation pathways after transferred ssc-miR-143 mimics; (B) The expression levels of key genes in the potential regulation pathways after transferred ssc-miR-143 inhibitor. (*p<0.05, n = 5).

To find out which pathway play a major role in the regulation of muscle fibres by ssc-miR-143-3p, we transferred ssc-miR-143-3p mimics and inhibitors into porcine skeletal muscle satellite cells, and detected some key genes in the potential regulation pathways (Fig 6), including *HDAC4*, *MEF2*, *MyoD* and *FLNC*. Results showed in Fig 7. The expression level of *HDAC4* in porcine skeletal muscle satellite cells was significantly deceased after transferred with ssc-miR-143 mimics (p<0.05). As *HDAC4* is a critical regulator of *MEF2*, the reduction of *HDAC4* significantly induce an increase of *MEF2* (p<0.05). At the same time, muscle satellite cells were transferred with ssc-miR-143 inhibitor. And the expression level of *HDAC4* and *MEF2* showed the opposite trends compared with transferred with ssc-miR-143 mimics. HDAC4 was significantly increased (p<0.05). And MEF2 was significantly decreased (p<0.05). However, the expression level of key genes *MyoD* and *FLNC* in the other two potential pathways have no changes both transferred with ssc-miR-143 mimics and inhibitor. The results all above indicated that the *HDAC4-MEF2* pathway play a crucial role in the muscle fibre regulation by ssc-miR-143.

## Materials and Methods

### Ethics statement

The animals were reared and slaughtered in strict compliance with the Welfare and Ethics of Laboratory Animals Regulations (WELAR) approved by the Chinese Association For Laboratory Animal Sciences (CAFLAS). All animal procedures were conducted under the protocol (SCAU-AEC-2010-0416) approved by the Institutional of Animal Ethics Committee (IAEC) of South China Agricultural University.

### Animals and muscle sampling

Sixty castrated hybrid male pigs (Landrace × Large White × Duroc) were used in this study. All animals were purchased from the experimental animal centre of the WENs Group. The average weight of the pigs at the beginning of the experiment was 7.27±0.13 kg. The animals were divided into 6 stalls with 10 animals per stall (3.5m×6m). All animals were housed in a controlled environment and were provided free access to food and water. The experimental diet was designed according to the NRC 2012 (nutrient requirements of swine, eleventh revised edition, 2012) as follows: 10–30 kg stage: 19.2% CP, 1.02% available lysine, and 3300 Kcal of DE/kg; 30–60 kg stage: 17.2% CP, 0.85% available lysine, and 3300 Kcal of DE/kg; and 60–110 kg stage: 15.2% CP, 0.69% available lysine, and 3300 Kcal of DE/kg. The experiment began when the pigs were 35 d old followed by a 5-d period to adapt to the new diet.

We slaughtered 18 pigs at 63, 98 and 161 d of age by slaughtering one pig from each stall. During the night before slaughter, the pigs were allowed ad libitum access to water but not food. The following morning, pigs were slaughtered by electrical stunning and exsanguination [47]. The psoas major muscle was then isolated. Within 1 h after slaughter, muscle samples were taken, mounted on tongue depressors, placed in freezing tubes, and stored in liquid nitrogen before transfer to a −80°C freezer.

### Histological analyses

Transverse serial sections of all muscle samples were generated using a cryostat (Leica Microsystems Nussloch GmbH, Leica CM 1850, Germany; set at 10 μm) at -20°C [48]. We integrated mATPase histochemistry with succinate dehydrogenase (SDH) histochemistry. The sections were stained for 45 min using SDH histochemistry after pre-incubation at pH 4.35 [4]. The sections were then washed and stained for 30 min using mATPase histochemistry [49]. Images of all sections were captured using a CCD camera connected to an optical microscope, and image analysis software was used to identify the fibre type. The fibres were classified into MyHC types 1, 2a and 2x+2b. We made 5 sections for each pig, and we selected 3 fields of view from each section for counting and calculations. All of the images were taken at 100X magnification.

### Total RNA extraction

Total RNA was isolated from the three stages (63d, 98d and 161d) psoas major muscle samplesusing TRIzol according to the manufacturer’s instructions. The RNA samples were treated with DNase to eliminate trace genomic DNA contamination. The total RNA was quantified by measuring the absorbance at 260 nm. The 260/280 nm and 260/230 nm absorbance ratios were measured to determine the purity of the isolated RNA. The integrity of the extracted RNA was determined by examining the 28S and 18S rRNA bands on ethidium bromide-stained agarose gels [50].

### Real-time quantitative PCR of MyHC genes

Using the total RNA as template and oligo (dt)18 as a primer, reverse transcription reactions were performed to generate cDNA using reverse transcriptase and an RNase inhibitor. The cDNAs of the muscle samples were stored at −80°C until use.

Oligo 7 and Primer Premier 5 were used to design primers to amplify the MyHC isoform genes and a housekeeping gene for RT-qPCR. Because the MyHC isoform genes belong to a single family, their sequences are highly similar. To amplify specific PCR products, we compared the gene sequences and designed primers annealing to regions with low similarity [47]. The primer sequences and PCR conditions used are listed in S8 Table. The RT-qPCR reactions were performed on an ABI 7500 machine using the cDNA as template and the SYBR Green fluorescent dye. The thermal cycling parameters were as follows: an initial denaturation step at 95°C for 10 min followed by 40 cycles of denaturation at 95°C for 15 s and annealing and extension at 60°C for 1 min. The expression levels of various MyHC mRNAs are reported as 2-ΔCt where ΔCt is the difference in Ct between MyHC and GAPDH. The relative amounts of MyHC 1, 2a, 2x and 2b are reported as percentage of the total MyHC transcripts.

### Real-time quantitative PCR for miRNAs

Reverse transcriptase reactions contained 1 μg of purified total RNA, 50 nM stem-loop RT primer (S8 Table), 10×RT buffer, 0.25 mM dNTP, 3.33 U/μl M-MLV reverse transcriptase and 0.25 U/μl RNase inhibitor [51]. The 7.5 μl reactions were incubated for 60 min at 42°C, 10 min at 75°C and then held at 4°C[52]. Real-time quantitative PCR was performed with standard protocols of the ABI 7500 system and using the reverse transcriptase reaction products as templates. The RT-qPCR thermal cycling parameters used are described in section 3.3. All reactions were run in duplicate and including negative controls without template.

### miRNA library preparation and Illumina deep sequencing

For the three developmental stages, equal quantities of total RNA isolated from six individual pigs psoas major muscle were pooled. The small RNA libraries of three stages were prepared according to the following steps. First, we sequentially ligated 3’ and 5’ adapters to the extracted total RNA using T4 RNA Ligase 2. Second, reverse transcription of small RNA was performed by PCR to ligate the small RNA to 3’ and 5’ adapters, and the cDNA was then amplified. Third, the cDNA construct was gel purified, and the library was validated according to a series of quality control analyses. The purified cDNA was then used for the Illumina deep sequencing analysis following the standard procedure of our service provider (LC Science).

### Statistical analysis

All data, including the *MyHC* evaluation, RT-qPCR validation and correlation coefficients, were analysed using SPSS 19.0. The results are presented as the least square means with standard errors. The differences between the measured quantities were analyzed using the T-test; p<0.05 was considered to represent a significant difference. Pearson correlation coefficients were determined using partial correlation coefficients to analyse the correlation between the two methods, RT-qPCR and deep sequencing.

The raw sequence data were processed using a proprietary pipeline script, ACGT101-miR v4.2 (LC Sciences), and a series of digital filters were employed to remove various unmappable sequencing reads. Various “mappings” were performed on unique sequences against pre-miRNA (mir) and mature miRNA (miR) sequences listed in miRBase Release 20.0 or genome based on the public releases of appropriate species. The unique sequences were achieved and used for subsequent analysis. All the sequence data have been submitted to the NCBI Gene Expression Omnibus (http://www.ncbi.nlm.nih.gov/geo/) under accession NO.GSE64275.

## Conclusions

In the period between 63 and 161 d, the ratio of slow-twitch oxidative fibres (type 1) and fast-twitch oxidative fibres (type 2a) decreased, revealing that the oxidation capacity of the psoas major muscle decreased during this period. Using Illumina deep sequencing, 271 mature miRNAs and 243 pre-miRNAs were identified in the psoas major muscle by comparing the sequences to the current miRBase database, and 472 novel miRNAs were detected in the muscle samples. The sequencing data were confirmed by RT-qPCR, and in highly similar sequences were generated from the two techniques. A comprehensive miRNA expression profile of the psoas major muscle in the period between 63 and 161 d has been established, which provides fundamental information about the miRNA regulation of muscle growth and differentiation. We found 23 highly expressed miRNAs in the miRNA expression profile analysis of these three stages. Among them, there are 10 muscle-related miRNAs, which control muscle fibre content, myofibre identity and muscle performance. We also discovered several miRNAs, including ssc-miR-1, ssc-miR-143-3p, ssc-miR-151, ssc-miR-30b, ssc-miR-340, and ssc-miR-335, that may be involved in fibre type regulation. Furthermore, overexpression/inhibition of ssc-miR-143-3p in SCs induced an/a increase/reduction of the slow muscle fibre gene and protein (*MYH7*), indicating that miR-143 activity regulated slow muscle fibre differentiate. MiRNAs in skeletal muscle play important roles in the process of muscle growth and differentiation suggesting a role for miRNAs in muscle fibre regulation and ultimately in muscle development.

## Supporting Information

S1 Fig Muscle fibre identified by histochemical staining. (DOCX)

S1 Table Overview of reads from raw data to cleaned sequences. (XLSX)

S2 Table Known porcine miRNAs identified in the psoas major muscle. (XLSX)

S3 Table The distribution of numbers for normalized miRNAs. (XLSX)

S4 Table Novel porcine miRNAs identified in the psoas major muscle via Solexa sequencing. (XLSX)

S5 Table miRNAs for KEGG pathway analysis. (XLSX)

S6 Table KEGG pathway enriched analysis for the targets of DHE miRNAs between different stages. (XLSX)

S7 Table MiRNAs and their target mRNAs with opposite expression trends. (XLSX)

S8 Table Primers for RT-qPCR. (XLSX)

## References

[1] Krawczynski K, Bauersachs S, Kaczmarek M. MicroRNA biogenesis-associated genes expression and miRNA expression profile in porcine embryos. Reprod Domest Anim. 2012;47:545–. PubMed PMID: WOS:000306918700414.

[2] Klont RE, Brocks L, Eikelenboom G. Muscle fibre type and meat quality. Meat science. 1998;49:S219–S29. PubMed PMID: WOS:000075885100017. 22060713

[3] Joo ST, Kim GD, Hwang YH, Ryu YC. Control of fresh meat quality through manipulation of muscle fiber characteristics. Meat science. 2013;95(4):828–36. 10.1016/j.meatsci.2013.04.044 . 23702339

[4] Lefaucheur L, Ecolan P, Plantard L, Gueguen N. New insights into muscle fiber types in the pig. Journal Of Histochemistry & Cytochemistry. 2002;50(5):719–30. PubMed PMID: WOS:000175151700013. 11967283 10.1177/002215540205000513

[5] Pette D, Staron RS. Transitions of muscle fiber phenotypic profiles. Histochemistry and cell biology. 2001;115(5):359–72. . 11449884 10.1007/s004180100268

[6] Macfarlane LA, Murphy PR. MicroRNA: Biogenesis, Function and Role in Cancer. Current genomics. 2010;11(7):537–61. 10.2174/138920210793175895 21532838 PMC3048316

[7] Valdez G, Heyer MP, Feng GP, Sanes JR. The Role of Muscle microRNAs in Repairing the Neuromuscular Junction. PloS one. 2014;9(3). doi: ARTN e93140 10.1371/journal.pone.0093140 PubMed PMID: WOS:000333459900157. PMC3963997 24664281

[8] van Rooij E, Quiat D, Johnson BA, Sutherland LB, Qi XX, Richardson JA, et al A Family of microRNAs Encoded by Myosin Genes Governs Myosin Expression and Muscle Performance. Developmental cell. 2009;17(5):662–73. 10.1016/j.devcel.2009.10.013 PubMed PMID: WOS:000272100700011. 19922871 PMC2796371

[9] Wells L, Edwards KA, Bernstein SI. Myosin heavy chain isoforms regulate muscle function but not myofibril assembly. The EMBO journal. 1996;15(17):4454–9. 8887536 PMC452174

[10] Jiang H, Jordan T, Li J, Li H, DiMario JX. Innervation-dependent and fiber type-specific transcriptional regulation of the slow myosin heavy chain 2 promoter in avian skeletal muscle fibers. Developmental dynamics: an official publication of the American Association of Anatomists. 2004;231(2):292–302. 10.1002/dvdy.20137 . 15366006

[11] Chen Z, Liang S, Zhao Y, Han Z. miR-92b regulates Mef2 levels through a negative-feedback circuit during Drosophila muscle development. Development. 2012;139(19):3543–52. 10.1242/dev.082719 22899845 PMC3436111

[12] Wang YX, Zhang CL, Yu RT, Cho HK, Nelson MC, Bayuga-Ocampo CR, et al Regulation of muscle fiber type and running endurance by PPARdelta. PLoS biology. 2004;2(10):e294 10.1371/journal.pbio.0020294 15328533 PMC509410

[13] el Azzouzi H, Leptidis S, Dirkx E, Hoeks J, van Bree B, Brand K, et al The hypoxia-inducible microRNA cluster miR-199a approximately 214 targets myocardial PPARdelta and impairs mitochondrial fatty acid oxidation. Cell metabolism. 2013;18(3):341–54. 10.1016/j.cmet.2013.08.009 . 24011070

[14] Handel SE, Stickland NC. The growth and differential of porcine skeletal muscle fiber types and the influence of birthweight. Journal of anatomy. 1987;152(5):107–19. 2958439 PMC1261750

[15] Wegner J, Albrecht E, Fiedler I, Teuscher F, Papstein HJ, Ender K. Growth- and breed-related changes of muscle fiber characteristics in cattle. Journal of animal science. 2000;78(6):1485–96. . 10875630 10.2527/2000.7861485x

[16] Ashmore CR, Tompkins G, Doerr L. Postnatal development of muscle fiber types in domestic animals. Journal of animal science. 1972;34(1):37–41. . 4258174 10.2527/jas1972.34137x

[17] Beatty CH, Peterson RD, Bocek RM. Metabolism of red and white muscle fiber groups. The American journal of physiology. 1963;204:939–42. . 13969982 10.1152/ajplegacy.1963.204.5.939

[18] Aguilar HN, Mitchell BF. Physiological pathways and molecular mechanisms regulating uterine contractility. Hum Reprod Update. 2010;16(6):725–44. 10.1093/humupd/dmq016 PubMed PMID: WOS:000283123200014. 20551073

[19] Gea J, Hamid Q, Czaika G, Zhu E, Mohan-Ram V, Goldspink G, et al Expression of myosin heavy-chain isoforms in the respiratory muscles following inspiratory resistive breathing. American journal of respiratory and critical care medicine. 2000;161(4 Pt 1):1274–8. 10.1164/ajrccm.161.4.99040103 . 10764323

[20] Qin L, Chen Y, Liu X, Ye S, Yu K, Huang Z, et al Integrative analysis of porcine microRNAome during skeletal muscle development. PloS one. 2013;8(9):e72418 10.1371/journal.pone.0072418 24039761 PMC3770649

[21] Mullokandov G, Baccarini A, Ruzo A, Jayaprakash AD, Tung N, Israelow B, et al High-throughput assessment of microRNA activity and function using microRNA sensor and decoy libraries. Nature methods. 2012;9(8):840–6. 10.1038/nmeth.2078 22751203 PMC3518396

[22] Li G, Li Y, Li X, Ning X, Li M, Yang G. MicroRNA identity and abundance in developing swine adipose tissue as determined by Solexa sequencing. Journal of cellular biochemistry. 2011;112(5):1318–28. 10.1002/jcb.23045 . 21312241

[23] Jordan T, Jiang H, Li H, DiMario JX. Regulation of skeletal muscle fiber type and slow myosin heavy chain 2 gene expression by inositol trisphosphate receptor 1. J Cell Sci. 2005;118(Pt 10):2295–302. 10.1242/jcs.02341 . 15870113

[24] Chen JF, Mandel EM, Thomson JM, Wu Q, Callis TE, Hammond SM, et al The role of microRNA-1 and microRNA-133 in skeletal muscle proliferation and differentiation. Nature genetics. 2006;38(2):228–33. 10.1038/ng1725 16380711 PMC2538576

[25] Zierath JR, Hawley JA. Skeletal muscle fiber type: influence on contractile and metabolic properties. PLoS biology. 2004;2(10):e348 10.1371/journal.pbio.0020348 15486583 PMC521732

[26] Small EM, O'Rourke JR, Moresi V, Sutherland LB, McAnally J, Gerard RD, et al Regulation of PI3-kinase/Akt signaling by muscle-enriched microRNA-486. Proceedings of the National Academy of Sciences of the United States of America. 2010;107(9):4218–23. 10.1073/pnas.1000300107 20142475 PMC2840099

[27] Bonauer A, Carmona G, Iwasaki M, Mione M, Koyanagi M, Fischer A, et al MicroRNA-92a controls angiogenesis and functional recovery of ischemic tissues in mice. Science. 2009;324(5935):1710–3. 10.1126/science.1174381 . 19460962

[28] Muroya S, Taniguchi M, Shibata M, Oe M, Ojima K, Nakajima I, et al Profiling of differentially expressed microRNA and the bioinformatic target gene analyses in bovine fast- and slow-type muscles by massively parallel sequencing. Journal of animal science. 2012;91(1):90–103. 10.2527/jas.2012-5371 23100578

[29] Jin W, Grant JR, Stothard P, Moore SS, Guan LL. Characterization of bovine miRNAs by sequencing and bioinformatics analysis. BMC molecular biology. 2009;10:90 10.1186/1471-2199-10-90 19758457 PMC2761914

[30] Takawa M, Cho HS, Hayami S, Toyokawa G, Kogure M, Yamane Y, et al Histone lysine methyltransferase SETD8 promotes carcinogenesis by deregulating PCNA expression. Cancer research. 2012;72(13):3217–27. 10.1158/0008-5472.CAN-11-3701 . 22556262

[31] Zhao X, Sternsdorf T, Bolger TA, Evans RM, Yao TP. Regulation of MEF2 by histone deacetylase 4- and SIRT1 deacetylase-mediated lysine modifications. Molecular and cellular biology. 2005;25(19):8456–64. 10.1128/MCB.25.19.8456-8464.2005 16166628 PMC1265742

[32] Murgia M, Serrano AL, Calabria E, Pallafacchina G, Lømo T, Schiaffino S. Ras is involved in nerve-activity-dependent regulation of muscle genes. Nature Cell Biology. 2000;2(3):142–7. 10707084 10.1038/35004013

[33] Rangrez AY, Massy ZA, Metzinger-Le Meuth V, Metzinger L. miR-143 and miR-145: molecular keys to switch the phenotype of vascular smooth muscle cells. Circulation Cardiovascular genetics. 2011;4(2):197–205. 10.1161/CIRCGENETICS.110.958702 . 21505201

[34] Esau C, Kang X, Peralta E, Hanson E, Marcusson EG, Ravichandran LV, et al MicroRNA-143 regulates adipocyte differentiation. The Journal of biological chemistry. 2004;279(50):52361–5. 10.1074/jbc.C400438200 . 15504739

[35] Chen L, Wu P, Guo XH, Hu Y, Li YL, Shi J, et al miR-143: A Novel Regulator of MyoD Expression in Fast and Slow Muscles of Siniperca chuatsi. Curr Mol Med. 2014;14(3):370–5. PubMed PMID: WOS:000333339100007. 24588768 10.2174/1566524014666140228100250

[36] Russell AP, Feilchenfeldt J, Schreiber S, Praz M, Crettenand A, Gobelet C, et al Endurance training in humans leads to fiber type-specific increases in levels of peroxisome proliferator-activated receptor-γ coactivator-1 and peroxisome proliferator-activated receptor-α in skeletal muscle. Diabetes. 2003;52(12):2874–81. 14633846 10.2337/diabetes.52.12.2874

[37] Golding JP, Calderbank E, Partridge TA, Beauchamp JR. Skeletal muscle stem cells express anti-apoptotic ErbB receptors during activation from quiescence. Experimental cell research. 2007;313(2):341–56. PubMed PMID: WOS:000243539000012. 17123512 10.1016/j.yexcr.2006.10.019

[38] Lebrasseur NK, Cote GM, Miller TA, Fielding RA, Sawyer DB. Regulation of neuregulin/ErbB signaling by contractile activity in skeletal muscle. Am J Physiol-Cell Ph. 2003;284(5):C1149–C55. PubMed PMID: WOS:000181982400007. 12519750 10.1152/ajpcell.00487.2002

[39] Bodine SC, Stitt TN, Gonzalez M, Kline WO, Stover GL, Bauerlein R, et al Akt/mTOR pathway is a crucial regulator of skeletal muscle hypertrophy and can prevent muscle atrophy in vivo. Nature cell biology. 2001;3(11):1014–9. 11715023 10.1038/ncb1101-1014

[40] Fazius F, Shelest E, Gebhardt P, Brock M. The fungal α-aminoadipate pathway for lysine biosynthesis requires two enzymes of the aconitase family for the isomerization of homocitrate to homoisocitrate. Molecular Microbiology. 2012;86(6):1508–30. 10.1111/mmi.12076 23106124 PMC3556520

[41] Wang L, Sahlin K. The effect of continuous and interval exercise on PGC-1alpha and PDK4 mRNA in type I and type II fibres of human skeletal muscle. Acta physiologica (Oxford, England). 2012;204(4):525–32. 10.1111/j.1748-1716.2011.02354.x . 21883960

[42] Wende AR, Huss JM, Schaeffer PJ, Giguere V, Kelly DP. PGC-1alpha coactivates PDK4 gene expression via the orphan nuclear receptor ERRalpha: a mechanism for transcriptional control of muscle glucose metabolism. Molecular and cellular biology. 2005;25(24):10684–94. 10.1128/MCB.25.24.10684-10694.2005 16314495 PMC1316952

[43] Dalkilic I, Schienda J, Thompson TG, Kunkel LM. Loss of FilaminC (FLNc) results in severe defects in myogenesis and myotube structure. Molecular and cellular biology. 2006;26(17):6522–34. 10.1128/MCB.00243-06 16914736 PMC1592847

[44] Zhao X, Sternsdorf T, Bolger TA, Evans RM, Yao T-P. Regulation of MEF2 by histone deacetylase 4-and SIRT1 deacetylase-mediated lysine modifications. Molecular and cellular biology. 2005;25(19):8456–64. 16166628 10.1128/MCB.25.19.8456-8464.2005 PMC1265742

[45] Wu H, Naya FJ, McKinsey TA, Mercer B, Shelton JM, Chin ER, et al MEF2 responds to multiple calcium-regulated signals in the control of skeletal muscle fiber type. The EMBO journal. 2000;19(9):1963–73. 10.1093/emboj/19.9.1963 10790363 PMC305686

[46] Ehlers ML, Celona B, Black BL. NFATc1 Controls Skeletal Muscle Fiber Type and Is a Negative Regulator of MyoD Activity. Cell reports. 2014;8(6):1639–48. PubMed PMID: WOS:000343867400005. 10.1016/j.celrep.2014.08.035 25242327 PMC4180018

[47] Lefaucheur L, Milan D, Ecolan P, Le Callennec C. Myosin heavy chain composition of different skeletal muscles in Large White and Meishan pigs. Journal of animal science. 2004;82(7):1931–41. PubMed PMID: WOS:000222317500006. 15309939 10.2527/2004.8271931x

[48] Larzul C, Lefaucheur L, Ecolan P, Gogue J, Talmant A, Sellier P, et al Phenotypic and genetic parameters for longissimus muscle fiber characteristics in relation to growth, carcass, and meat quality traits in large white pigs. Journal of animal science. 1997;75(12):3126–37. . 9419985 10.2527/1997.75123126x

[49] Nachlas MM, Tsou KC, De Souza E, Cheng CS, Seligman AM. Cytochemical demonstration of succinic dehydrogenase by the use of a new p-nitrophenyl substituted ditetrazole. The journal of histochemistry and cytochemistry: official journal of the Histochemistry Society. 1957;5(4):420–36. . 13463314 10.1177/5.4.420

[50] Wimmers K, Ngu NT, Jennen DG, Tesfaye D, Murani E, Schellander K, et al Relationship between myosin heavy chain isoform expression and muscling in several diverse pig breeds. Journal of animal science. 2008;86(4):795–803. 10.2527/jas.2006-521 . 18156349

[51] Chen C, Ridzon DA, Broomer AJ, Zhou Z, Lee DH, Nguyen JT, et al Real-time quantification of microRNAs by stem–loop RT–PCR. Nucleic acids research. 2005;33(20):e179–e. 16314309 10.1093/nar/gni178 PMC1292995

[52] Li HY, Xi QY, Xiong YY, Cheng X, Qi Q, Yang L, et al A Comprehensive Expression Profile of MicroRNAs in Porcine Pituitary. PloS one. 2011;6(9). doi: ARTN e24883 10.1371/journal.pone.0024883 PubMed PMID: WOS:000295936900017. PMC3182167 21969866

