# Supplementary figures and images for: MicroRNA Transcriptome Profile Analysis in Porcine Muscle and the Effect of miR-143 on the MYH7 Gene and Protein
Source: PLoS One. 2015 Apr 27;10(4):e0124873. doi: 10.1371/journal.pone.0124873 (PMC4410957; doi:10.1371/journal.pone.0124873)

**S1 Fig.** **Muscle fibre identified by histochemical staining.**


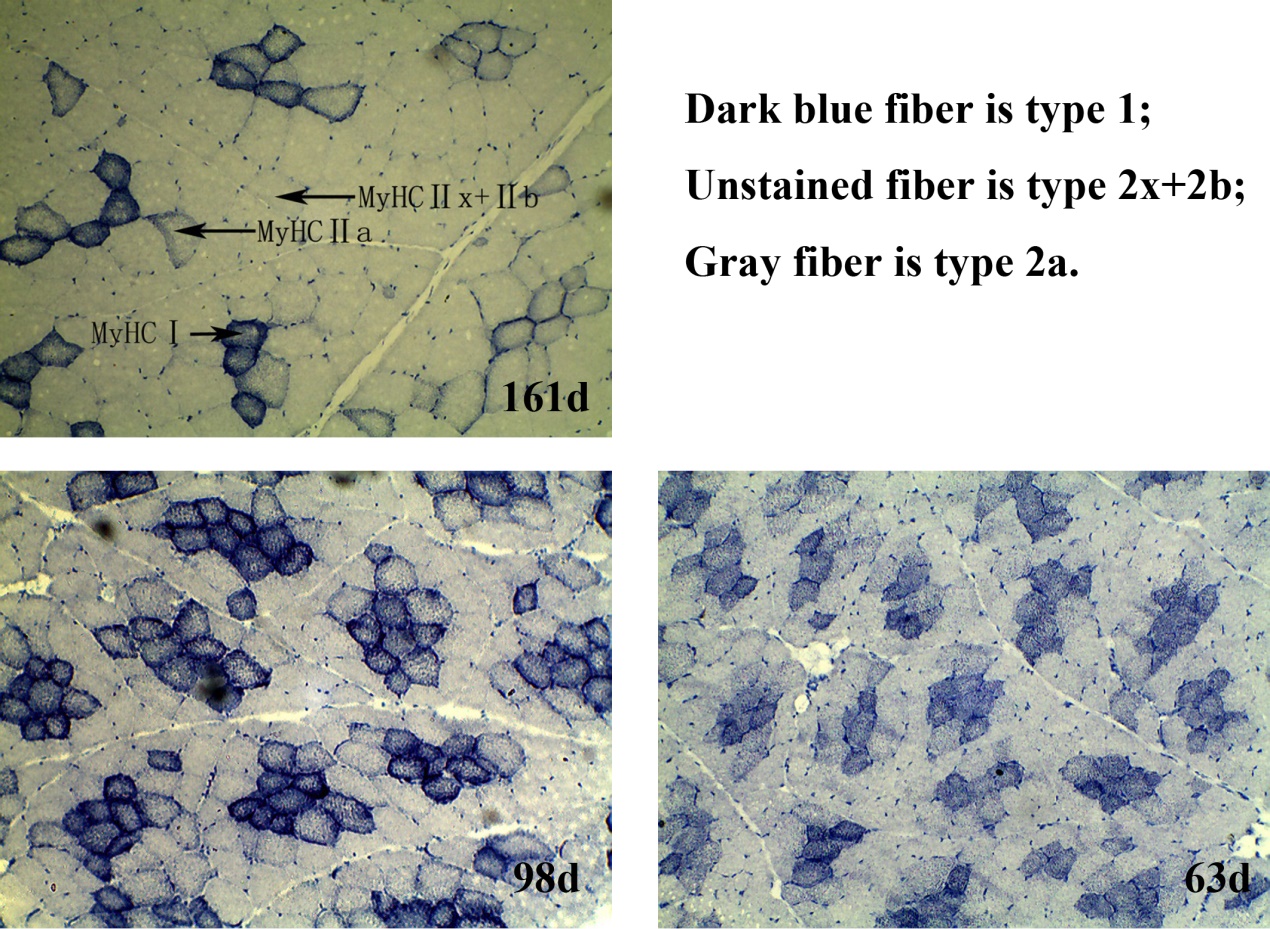

Supplement: S1 Fig — (DOCX) [file pone.0124873.s001.docx]
